# Perennially young: seed production and quality in controlled and natural populations of *Cistus albidus* reveal compensatory mechanisms that prevent senescence in terms of seed yield and viability

**DOI:** 10.1093/jxb/ert372

**Published:** 2013-11-11

**Authors:** Maren Müller, Laura Siles, Jana Cela, Sergi Munné-Bosch

**Affiliations:** Departamento de Biologia Vegetal, Facultat de Biologia, Universitat de Barcelona, Avinguda Diagonal 643, 08028 Barcelona, Spain

**Keywords:** Ageing, *Cistus albidus* L., perennials, seed composition, seed production, senescence, vitamin E.

## Abstract

The question of whether or not perennial plants senesce at the organism level remains unresolved. The aim of this study was to unravel whether or not plant age can influence the production and composition of seeds. Flower and seed production was examined in 3-, 8-, and 13-year-old *Cistus albidus* plants growing in experimental plots corresponding to the F_2_, F_1_, and F_0_ generations of the same population. Furthermore, the phytohormone, fatty acid, and vitamin E content of the seeds was evaluated, and their viability was examined. Whether or not age-related differences in seed quality were observed in a natural population in the Montserrat Mountains (NE Spain) was also tested. The results indicate that under controlled conditions, the oldest plants not only produced fewer flowers, but also had higher rates of embryo abortion in mature seeds. However, germination capacity was not negatively affected by plant ageing. Seeds of the oldest plants contained significantly higher salicylic acid, jasmonic acid, and vitamin E levels compared with those from younger plants. Despite vigour (in terms of plant growth) being severely reduced due to harsh environmental conditions in the natural population, the oldest individuals produced seeds with no decline in viability. Seed biomass was instead positively correlated with seed viability. In conclusion, increased plant size may explain the loss of seed viability in the experimental field, but older smaller individuals in natural populations can escape senescence in terms of seed viability loss.

## Abstract (precis)

This study evaluated if plant ageing can influence the production and composition of seeds in controlled and natural populations of *Cistus albidus*. Results indicate that reduced plant size in natural populations can help old individuals escape senescence in terms of seed viability loss

## Introduction

It is well documented that annual plants enter a controlled senescence programme. In most cases, this is associated with flowering in monocarpic plants (annuals, biennials, and some perennials with a single reproductive episode). However, it is a matter of current debate as to whether or not iteroparous perennial plants, such as trees and shrubs, senesce (Munné-Bosch, 2008; Wingler *et al.*, 2009; Peñuelas and Munné-Bosch, 2010; de Witte and Stöcklin, 2010; Thomas, 2013). While some recent studies have shown symptoms of senescence at the whole-plant level in iteroparous perennials (Ally *et al.*, 2010; Herrera and Jovani, 2010), other studies failed to report senescence symptoms with plant ageing (García *et al.*, 2011; Morales *et al.*, 2013). These results present new challenges for general theories of biological ageing, as they question whether or not ageing of living organisms is a universal pattern in nature. The arguments against these theories are based, at least in part, on the fact that perennials maintain the capacity to develop new leaves and grow throughout their life, due to the presence of meristems. Unless damaged, meristems can potentially give rise to new structures throughout the entire life of a plant. Therefore, some individuals can live for millennia, even without using clonal growth (e.g. the oldest known bristlecone pine has been dated to >4500 years). However, have these organisms suffered age-associated physiological deterioration, or are they young, potentially immortal individuals?

Plants grow vigorously in the early stages of development, until a maximum height is attained at a certain age. Growth rates then tend to decline. With increasing age and size, relative leaf growth and photosynthetic rates in woody perennials tend to slow down. Increased size, rather than meristem ageing, has been proposed as a factor that determines age-related reductions in growth and photosynthetic rates in leaves (Mencuccini *et al.*, 2005; Vanderklein *et al.*, 2007; Oñate and Munné-Bosch, 2008). Similarly, flower production increases with size and total leaf area at early stages of plant development. When advanced developmental ages are reached, flower production becomes stable. For instance, the production of flowers increases with total leaf area in the herbaceous perennial *Corydalis intermedia*. It then reaches a plateau around an age of 11 years, after which the number of flowers produced remains constant (Ehlers and Olesen, 2004). This study suggests that, given a limited amount of resources, an individual plant allocates a fraction of its resources to reproduction and the remaining resources to survival and growth. Another study found that flower production in the herbaceous perennial *Potentilla recta* correlates much better with site elevation than with ageing (Perkins *et al.*, 2006). Furthermore, it has been shown that flower production remains constant in 5- to 10-year-old *C. albidus* plants, even though flower bud vigour decreases with age. This suggests symptoms of senescence at the whole-plant level (Oñate and Munné-Bosch, 2010). Unfortunately, studies of age-related changes in flower production in woody perennials (shrubs or trees) are limited, particularly in older individuals. This is partly because of the intrinsic limitation of such studies: as time elapses and plants age, so the mortality risk increases. To the authors’ knowledge, the effects of plant ageing on seed production in woody perennials has not yet been investigated.

The aim of the present study was to determine whether or not *C. albidus* plants show symptoms of senescence at the whole-plant level when individuals of sufficiently old ages are sampled. The focus was on senescence symptoms in seed production. Two populations were studied: the first under controlled conditions in experimental plots in which F_2_, F_1_, and F_0_ plants from the same population were assessed, while the second one was studied under natural conditions in the Montserrat Mountains (NE Spain).

## Materials and methods

### Plant material and sampling

*Cistus albidus* was selected as the experimental model. This is a common Mediterranean shrub that is widely distributed in the western Mediterranean from sea level to 1400 m (Blasco and Mateu, 1995). This shrub is resistant to drought stress and has a high capacity to grow in degraded environments. The life span of this species is thought to be ~15 years (Roy and Sonié, 1992), so it was possible to evaluate the effects of plant ageing by using plants at advanced developmental stages.

Two independent studies were performed. The first study was conducted using three groups of *C. albidus* plants of different ages (3-, 8-, and 13-year-old plants) in the Experimental Fields of the Faculty of Biology at the University of Barcelona (Barcelona, Catalonia, NE Spain) in 2011. All specimens were considered mature, since this plant becomes reproductive for the first time in the second year of life. The three plant groups corresponded to three consecutive generations of plants obtained under controlled conditions. The F_0_ generation (13-year-old plants) corresponded to individuals obtained from seeds that germinated during 1998 and were grown in 0.5 litre pots containing a mixture of soil:peat:perlite (1:1:1, v/v/v), maintained in a greenhouse with controlled temperature and watering for 1 year and then transferred to plots in the Experimental Fields, in which the plants grew for 12 years before the study began. The F_1_ and F_2_ generations (8- and 3-year-old plants) corresponded to individuals also sampled during 2011 but obtained from seeds of the preceding generation that germinated during 2008 and 2003, and were grown in 0.5 litre pots containing a mixture of soil:peat:perlite (1:1:1, v/v/v), maintained in a greenhouse with controlled temperature and watering for 1 year and then transferred to plots in the Experimental Fields, in which the plants grew for 7 years and 2 years, respectively, before the study began. Although not identical, due to annual variation, all three plant groups were exposed to Mediterranean climatic conditions during their life histories and were grown in three plots of calcic Luvisol (FAO) of exactly the same characteristics. Plants were separated by 2 m. They were not watered manually other than when first transferred to the soil, at which point all plant groups were watered equally and treated with N:P:K (1:1:1) fertilizer at a rate of 100kg ha^–1^. Fertilizer was applied again only when mineral deficiencies were detected (maximum application: once a year), but always equally to the three plant groups. All groups were therefore exposed to identical climatic and soil conditions, with the exception of the time spent in these conditions before sampling. Therefore, the effects of ageing were evaluated using three generations grown under the same climatic and soil conditions.

In a second experiment, a natural population of *C. albidus* was studied. Ninety-one individuals growing naturally in the Natural Park of the Montserrat Mountains (50 km northwest of Barcelona, Catalonia, NE Spain) were sampled. More specifically, the plants were found between 1000 m and 1149 m above sea level (UTM: 401.2012,4.606.724) on a north-facing site. The trunk perimeter of the sampled individuals ranged from 2cm to 22cm. The Montserrat Mountains are exposed to Mediterranean climatic conditions, but summers are drier and winters colder than at the Experimental Fields in Barcelona. The individuals sampled were on the sunny side of the mountain and the soil was a mixture of conglomerate, sandstone, and red shale. Mature seeds from both populations were obtained from plants during September 2011 in the Experimental Fields and in 2012 in the Montserrat Mountains, when fruits were fully mature.

### Age estimation

The trunk perimeter of all individuals growing in the Experimental Fields (for several years) and in the Natural Park (during 2012) was measured ~4cm from the trunk base with a measuring tape. Since the age of individuals growing in the Experimental Fields was already known (based on sowing time; see previous section) and the age of 15 individuals growing naturally in the Natural Park was estimated by counting the trunk rings, a regression of the trunk perimeter with the age of individuals was calculated.

### Flower and fruit production

The number of flowers produced per individual was counted every day during the flowering period (February–June) in plants growing in the Experimental Fields. Fruit biomass and number of seeds per fruit were estimated from 50 fruits per individual. The percentage of aborted fruits was also estimated by marking 50 flowers (at anthesis) per individual and evaluating the number of mature fruits formed. Fruit and seed biomass were also estimated by weighing the samples both in the Experimental Fields and under natural field conditions. The number of seeds per fruit was also counted.

### Seed germination and viability tests

Seed germination and viability tests were performed using seeds collected from both the Experimental Fields and natural field conditions. For the study conducted at the Experimental Fields, 250 seeds per age group were used (five subsamples of 50 seeds). Under natural field conditions, 50 seeds per individual were used.

Germination tests were carried out by sterilizing the seeds in an aqueous solution of bleach and Tween-20 (50:0.15, v/v) for 10min. The seeds were then imbibed for 24h in Milli Q water before being subjected to a heat shock (100 °C, 5min). The seeds were germinated at 17 °C. All steps of the germination test were performed in the dark.

Viability tests were performed as follows. Seeds were imbibed for 24h in the dark and a heat shock was applied as described above. The seeds were then soaked in a tetrazolium solution at 1% and incubated at 37 ºC in darkness for 2 d. Subsequently, seeds were bleached for 10min at 80% and the viable and non-viable seeds were counted. Seeds were considered to be alive when the embryo was intact and fully stained with rich formazan red. Seeds were considered to be non-viable when staining of the embryo was patchy, weak (pink), or absent. A fraction of these seeds were empty (aborted seeds).

### Vitamin E and hormonal profiling

The extraction and analyses of tocopherols and tocotrienols were performed as described by Cela *et al.* (2011). The extraction and analyses of endogenous concentrations of phytohormones, including gibberellins (GAs), abscisic acid (ABA), auxin [indole-3-acetic acid (IAA)], jasmonic acid (JA), salicylic acid (SA), and cytokinins, were performed as described by Müller and Munné-Bosch (2011).

### Elemental and fatty acid analyses

Total C and N concentrations as well as the fatty acid profile were measured in seeds of plants growing in the Experimental Fields. Elemental analyses were measured using the Dumas method and an NA2100 protein nitrogen analyser (Thermo, Milan, Italy). The extraction and analyses of fatty acids were performed as described by Vrinten *et al.* (2005).

### Statistical analyses

Differences between age groups were evaluated using analysis of variance (ANOVA), with DMS’s post-hoc test for plants grown in the Experimental Fields. Results were considered significant at a probability level of *P* ≤ 0.05. The significance of correlations between parameters measured in seeds obtained from plants growing in the Natural Park was tested using Spearman’s rank correlation analyses, and correlations were considered significant when *P* was ≤0.05.

## Results

### Flower, fruit, and seed production in the Experimental Fields

**Fig. 1.**
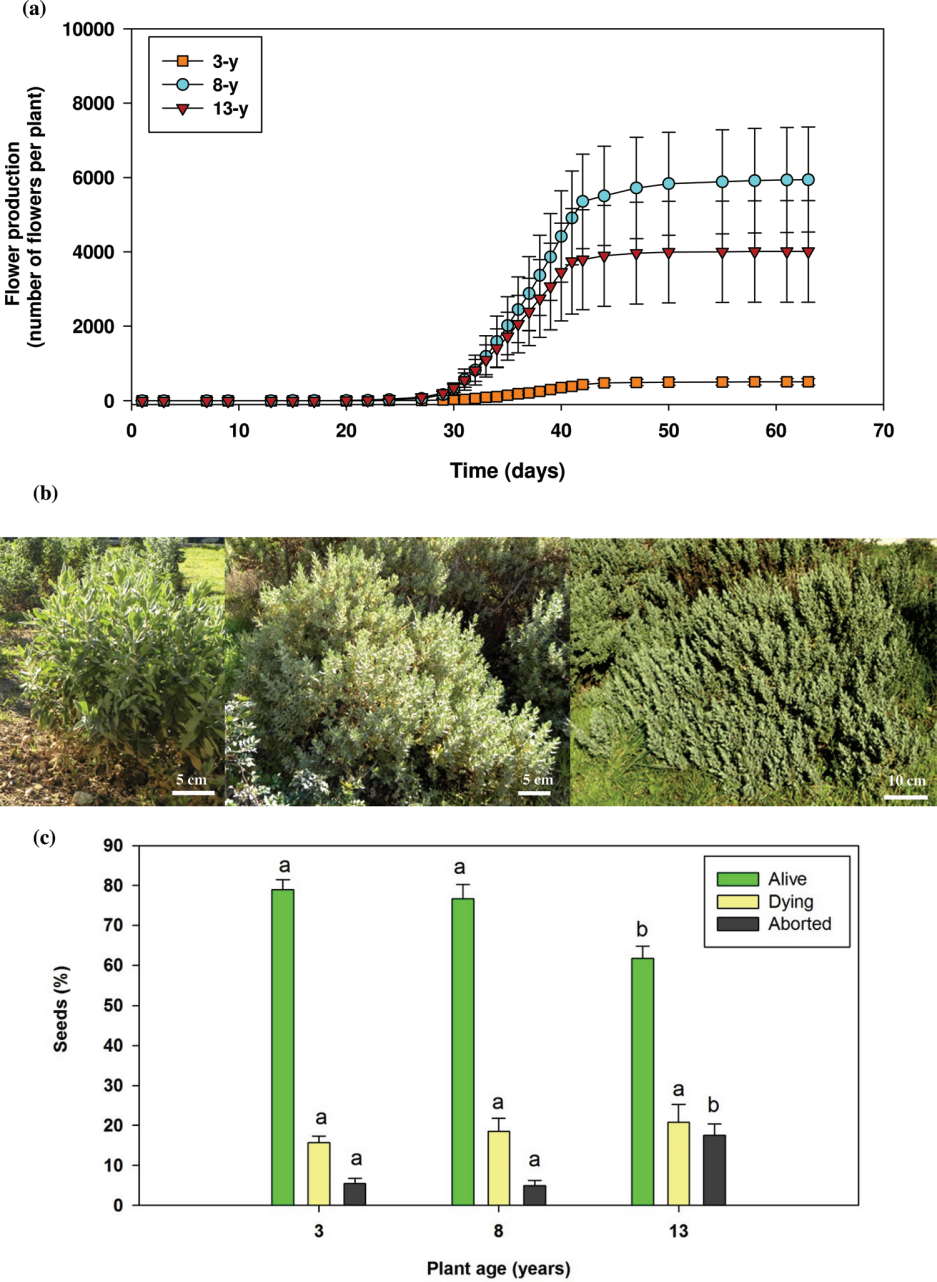
(a) Flower production (number of flowers) per individual in 3-, 8-, and 13-year-old *C. albidus* plants growing in the Experimental Fields. Time corresponds to days elapsed since the first flower was observed. Arrows indicate the average date of start of flowering in the three plant groups (24.3±1.5, 7.6±3.5, and 13.0±4.2 d for 3-, 8-, and 13-year-old plants, respectively). Data are the mean ±SE of *n*=16 individuals for 3-year-old plants and *n*=4 individuals for 8- and 13-year-old plants. Statistical analyses indicated significant differences between 3- and 8- or 13-year-old plants, but not between 8- and 13-year-old plants in either flower production or time of flowering (*P* ≤ 0.05). (b) Photographs of plants growing in the experimental fields. Left to right: 3-, 8-, and 13-year-old plants. (c) Percentage of live, dying, and aborted seeds during fruit production in 3-, 8-, and 13-year-old *C. albidus* plants. Data are the mean ±SE of *n*=4 individuals with an analysis of 50 seeds per individual. Different letters indicate significant differences between age groups (ANOVA, *P* ≤ 0.05). (This figure is available in colour at *JXB* online.)

Flower production was affected by plant age in the Experimental Fields. The highest rate of flower production was observed in 8-year-old plants, with an average of ~6000 flowers, followed by the oldest plant group with 4000 flowers, and the youngest plant group with 500 flowers per plant. The first flower was observed in the 8-year-old plants. The 13-year-old plants began to flower 6 d later and the smaller 3-year-old plants 16 d later (Fig. 1a). This timing was consistent with plant size, with the 13- and 8-year-old plants being of similar size, but much bigger than the 3-year-old plants (Fig. 1b). Therefore, reproduction was reduced and delayed in the youngest plant group. However, the high flower production in the two oldest plant groups seemed to affect the biomass and the number of seeds per fruit negatively (Table 1). Thirteen- and 8-year-old plants produced 16% and 40% lower fruit biomass and 31% and 53% fewer seeds per fruit, respectively, than the youngest plant group. Seed biomass decreased significantly with increasing plant age (Table 1). There were no differences between the 3- and 8-year-old plants in the number of alive, dying, and aborted seeds. However, 13-year-old plants produced 20% fewer viable seeds and 3-fold more aborted seeds than the younger plants (Fig. 1c). Nevertheless, comparison of the seed germination capacity revealed no significant variation between the three plant groups (Supplementary Fig. S1 available at *JXB* online).

**Table 1.** **F**ruit biomass, percentage of aborted fruits (from anthesis), number of seeds per fruit, and seed biomass in 3-, 8-, and 13-year-old C. albidus plants growing in the Experimental Fields

Plant age (years)	Fruit biomass (mg FW fruit^–1^)	Fruit abortion (%)	No. of seeds per fruit	Seed biomass (mg FW)
3	94.3±2.7 a	1.1±0.6 a	66.6±2.4 a	50.8±0.9 a
8	56.1±3.3 b	None	31.3±3.5 b	43.3±0.6 b
13	79.0±3.0 c	1.1±0.7 a	45.7±2.3 c	39.3±0.7 c

Data are the mean ±SE of *n*=4 with an analysis of 50 fruits per individual. Different letters indicate significant differences between age groups (*P* ≤ 0.05).

### Biochemical seed composition in the Experimental Fields

**Fig. 2.**
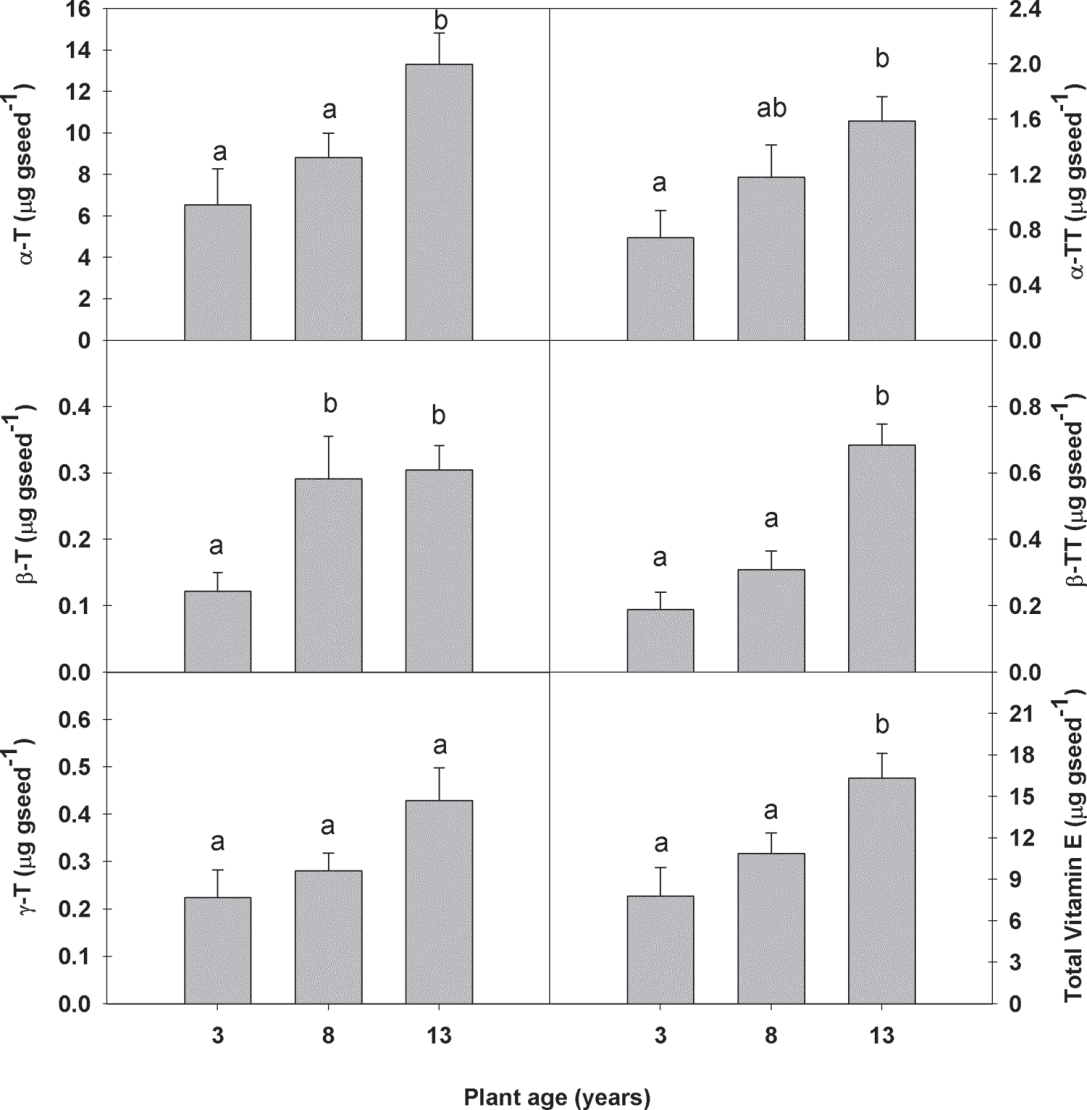
Tocopherol, tocotrienol, and total vitamin E content in seeds of 3-, 8-, and 13-year-old *C. albidus* plants growing in the Experimental Fields. Data are the mean ±SE of *n*=4 individuals with an analysis of 50mg of seeds per individual. Different letters indicate significant differences between age groups (ANOVA, *P* ≤ 0.05).

A screen of the fatty acid composition showed that the seeds of the oldest plant group had significantly higher total polyunsaturated fatty acid (PUFA) levels than the seeds from 3- and 8-year-old plants. This was mainly due to their higher levels of linoleic acid (C18:2) (Supplementary Table S1 at *JXB* online). The seeds of the oldest plant group also showed a tendency to synthesize higher concentrations of very long chain saturated fatty acids. However, only the concentrations of tetracosanoic acid (C24:0) were significantly higher (~17%) than in the seeds of the younger plant groups. Interestingly, the seeds of the oldest plant group had significantly higher total vitamin E levels at 17.6 μg (g DW)^–1^ than the seeds from 3- and 8-year-old plants, at 8.3 and 11.6 μg (g DW)^–1^, respectively (Fig. 2). The main vitamin E compound in *C. albidus* seeds was α-tocopherol. δ-Tocopherol, δ-tocotrienol, and γ-tocotrienol were not detected.

**Fig. 3.**
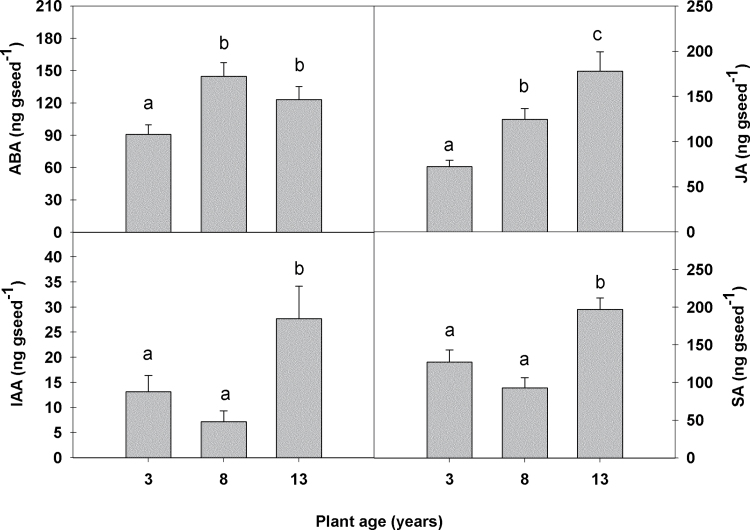
Abscisic acid (ABA), indole-3-acetic acid (IAA), jasmonic acid (JA), and salicylic acid (SA) content in seeds of 3-, 8-, and 13-year-old *C. albidus* plants growing in the Experimental Fields. Data are the mean ±SE of *n*=4 individuals with an analysis of 50mg of seeds per individual. Different letters indicate significant differences between age groups (ANOVA, *P* ≤ 0.05).

No significant variation in GA_4_ or the precursor GA_24_ was found between seeds from any of the plant groups. However, the concentration of the precursor GA_9_ was significantly lower in the seeds of 3-year-old plants (Supplementary Fig. S2 at *JXB* online). Endogenous concentrations of ABA were significantly higher in seeds from 8- and 13-year-old plants [154.6ng (g DW)^–1^ and 133.28ng (g DW)^–1^, respectively] than in seeds from 3-year-old plants [96.92ng (g DW)^–1^] (Fig. 3). However, JA levels also increased significantly with increasing plant age: seeds from 8- and 13-year-old plants contained 42% and 60% higher levels, respectively, than seeds from 3-year-old plants. In contrast, the IAA and SA content was only significantly higher in the seeds of the oldest plants (for IAA, 2- and 4-fold; and for SA, 1.58- and 2.15-fold higher than in seeds from 3- and 8-year-old plants, respectively). Within the cytokinins, no differences were found between the three plant age groups, with the exception of levels of zeatin (Z) (Supplementary Fig. S3). Z is the major cytokinin form found in *C. albidus* seeds, and was ~5-fold higher in seeds from 8-year-old plants than in seeds from 3-year-old plants. The Z levels found in seeds from 13-year-old plants did not differ significantly from those in the other two plant groups.

No significant variation in C and N concentrations or the C/N ratio was found in seeds of any plant group (Supplementary Fig. S4 at *JXB* online).

### Fruit and seed production in a natural population

**Fig. 4.**
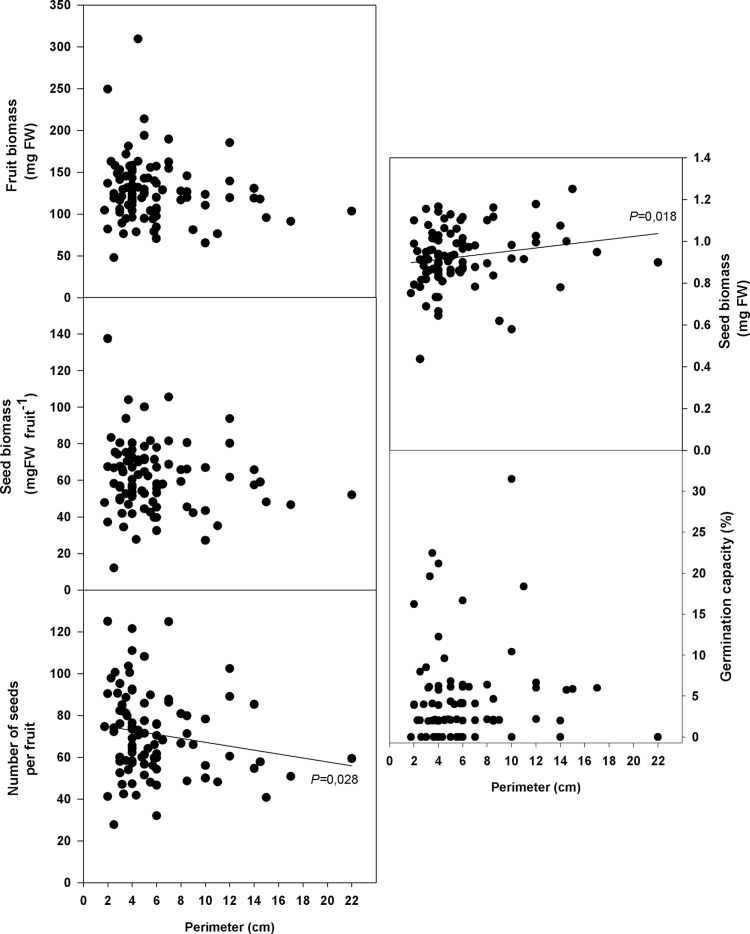
Fruit biomass, number of seeds per fruit, seed biomass per fruit, biomass of seeds, and seed germination capacity of *C. albidus* plants with different perimeters growing in a natural population in the Montserrat Mountains (NE Spain). *P*-values were calculated by Spearman’s rank correlation, and significant values (*P* ≤ 0.05) are shown.

**Fig. 5.**
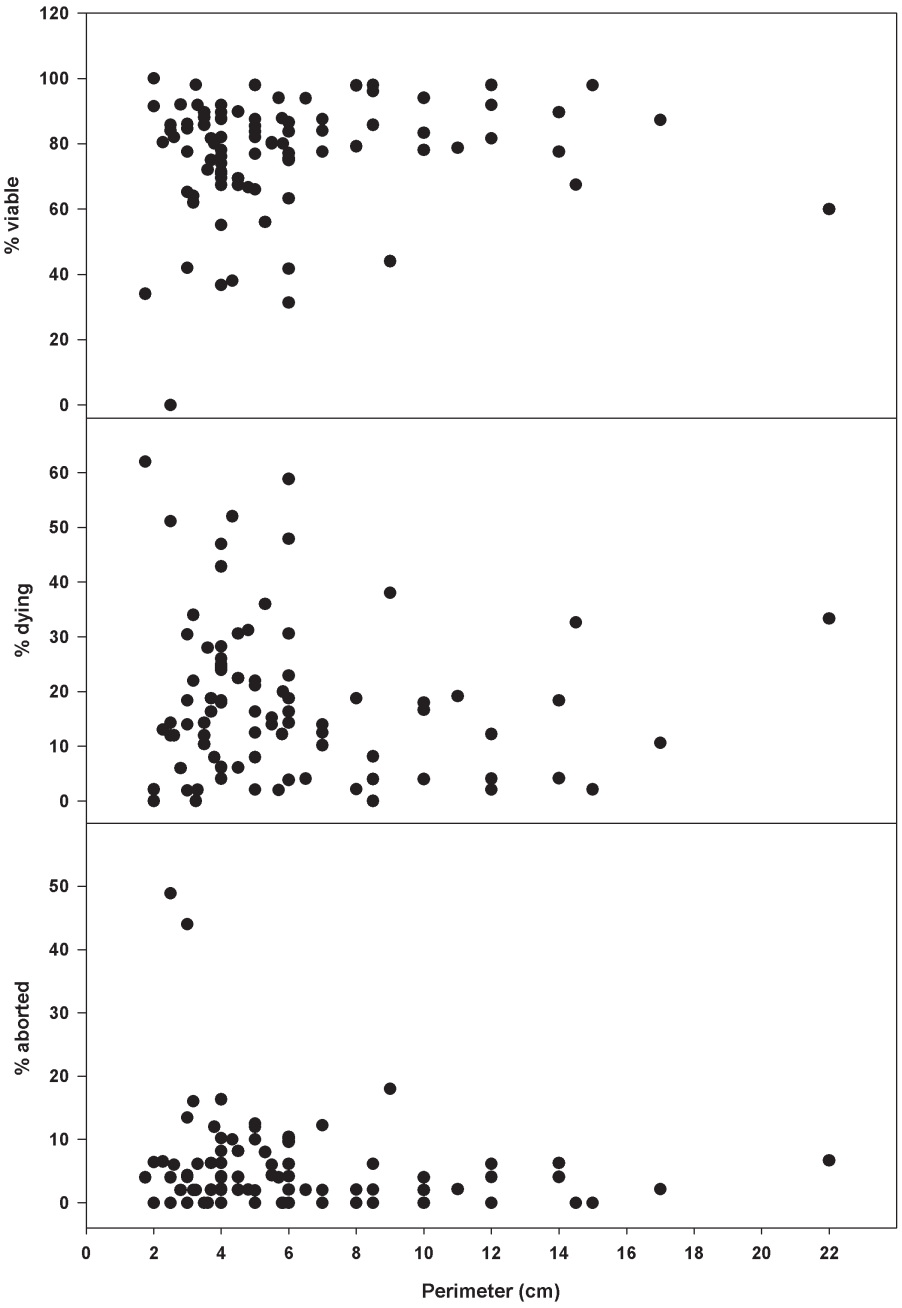
Percentage of live, dying, and aborted seeds of *C. albidus* plants with different perimeters growing in a natural population in the Montserrat Mountains (NE Spain). Spearman’s rank correlations were not significant for any studied parameter (*P* > 0.05).

**Fig. 6.**
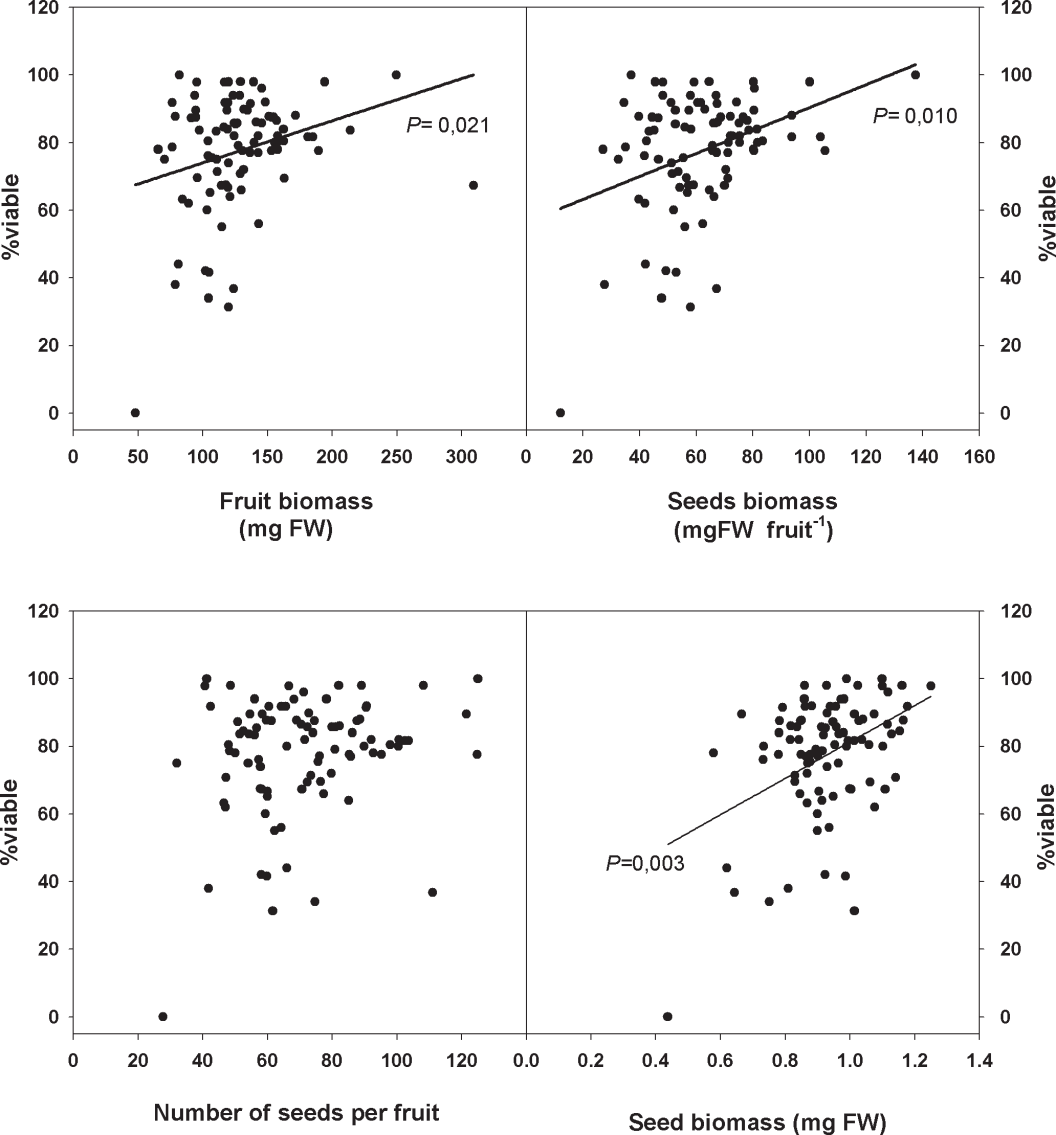
Correlation between seed viability and fruit biomass, seed biomass per fruit, number of seeds per fruit, and seed biomass in a natural population in the Montserrat Mountains (NE Spain). *P*-values were calculated by Spearman’s rank correlation, and significant values (*P* ≤ 0.05) are shown.

Fruit biomass and seed biomass per fruit were not correlated with the trunk perimeter of the individuals sampled in the Natural Park. In contrast, individuals with larger perimeters produced fewer seeds per fruit (from 79 down to 60 seeds) but larger seeds (from 0.9mg to 1mg fresh matter per seed; Fig. 4). All individuals (except one with a perimeter of 2.5cm) had viable seeds, most of them (75 from 85 individuals) with a seed viability ranging from 60% to 100% (Fig. 5). The percentage of dying seeds ranged between 0% and 60%, but in general the number of dying seeds was low. There were very few aborted seeds except for two individuals. Most importantly, seed viability was not correlated with trunk perimeter. In other words, since trunk perimeter positively correlated with plant ageing (Supplementary Fig. S5 at *JXB* online), seed viability was not negatively affected by plant age. Instead, seed viability was correlated with fruit biomass, seed biomass, and seed biomass per fruit (Fig. 6). The larger the fruits and seeds, the more viable the seeds produced by individuals in the natural population. Seed germination capacity, which was in general lower than in individuals grown under controlled conditions, was also not affected by plant age (Fig. 4).

### Biochemical seed composition in a natural population

The vitamin E profiling of seeds of *C. albidus* growing in the Montserrat Mountains confirmed that α-tocopherol is the major vitamin E compound in seeds. The amounts of tocopherols and tocotrienols were not correlated with the perimeter (Supplementary Table S2 at *JXB* online). δ-Tocopherol, γ-tocotrienol, and δ-tocotrienol were not detected. Likewise there was much variability in plant hormones between individuals with different perimeters and even between individuals with the same perimeter, and thus there was no correlation with perimeter or therefore with age. Levels of GA_4_, GA_9_, and GA_24_, ABA, auxin, SA, JA, and cytokinins were not correlated with the perimeter (Supplementary Table S2).

## Discussion

The life span of plants ranges from a few weeks for annuals to thousands of years for some trees (Bliss, 1971; Brundu *et al.*, 2008). Understanding the mechanisms underlying the wide range of longevity in plants or any other organism is fundamental to our understanding of life history, population dynamics, and evolutionary fitness (Vaupel *et al.*, 2004). For most annuals and biennials, reproduction is one of the key factors leading to whole-plant senescence. However, little is known about whole-plant senescence in perennials. To the authors’ knowledge, this is the first study to analyse the effect of mother plant age on seed production and quality in woody perennials.

The study performed in the Experimental Fields showed clear signs of senescence in the 13-year-old plants, in which flower production was 40% lower than in the 8-year-old plant group. The oldest plant group produced more seeds per fruit, but seed production per individual was 13% lower overall. This latter result is based on the assumption that flower vigour was similar in both plant groups, but this is not the case. In a previous study, it was shown that flower bud vigour declines with ageing (Oñate and Munné-Bosch, 2008); therefore, seed production per individual may be even more than 13% lower in 13-year-old compared with 8-year-old plants. Furthermore, seed abortion was higher in the oldest plant group in the Experimental Fields, therefore indicating senescence at the whole-plant level under controlled conditions. This is not surprising, since it has been well documented that when perennials reach an optimal plant size, plant ageing leads to a reduction in growth and photosynthetic rates in leaves (Bond, 2000; Koch *et al.*, 2004; Mencuccini *et al.*, 2005; Oñate and Munné-Bosch, 2008); however, evidence of reproductive senescence (i.e. reproductive decline with ageing) in non-clonal woody perennials is limited. In the perennial *Corydalis intermedia*, an increase in flower production and total leaf area was observed at early stages of plant development. Around the age of 11 years, flowering reached a plateau and the plants then produced a constant number of flowers (Ehlers and Olesen, 2004). Similar results were found for *C. albidus* plants, in which flower production was almost identical in 5- and 10-year-old plants of similar sizes (Oñate and Munné-Bosch, 2010). It has been suggested that maternal regulation of offspring quality (Stephenson, 1981) or genetic load (Stanton, 1984; Wiens *et al.*, 1987) are possible mechanisms for determining seed abortion when resources are limited. Intrafruit resource competition could also lead to seed reduction and arise from the fact that the maternal plant predominantly invests resources in zygotes that have higher probabilities of maturing (Shaanker *et al.*, 1988; De Jong and Klinkhamer, 2005). Therefore, it is not surprising that physiological deterioration occurs in seeds with ageing of the mother plant. However, seeds from 13-year-old plants showed similar germination capacity to seeds from the younger individuals. This suggests that a compensatory mechanism may enable similar reproductive fitness in plants growing in the Experimental Fields, such that the offspring are not affected. The higher levels of vitamin E in seeds from 13-year-old plants could explain the similar germination capacity of this group, as α-tocopherol is an important compound for germination (Sattler *et al.*, 2004). Seed viability and vigour are important aspects determining the success of seed germination, which is a key trait for the survival of a species (Nonogaki *et al.*, 2010), but, at the same time, seed germination is a very sophisticated process that requires the concerted action and interaction of diverse plant hormones (Kucera *et al.*, 2005). Although there were no differences in either GA or cytokinin content, the higher levels of ABA, JA, IAA, and SA indicate that an altered hormonal balance might be behind the improved germination capacity, despite the increased proportion of aborted seeds. JA and SA are associated with increased resistance to insects and fungi (Davies, 2010), respectively; therefore, increases in the levels of these phytohormones in seeds of the oldest plants could serve a compensatory mechanism and increase the survival of the seeds. Although a complete understanding of the complex hormonal cross-talk and signalling events leading to this phenotype remains elusive, the present results suggest that vitamin E and hormones may underlie the similar germination capacity despite greater embryo abortion in the oldest plant group.

**Fig. 7:**
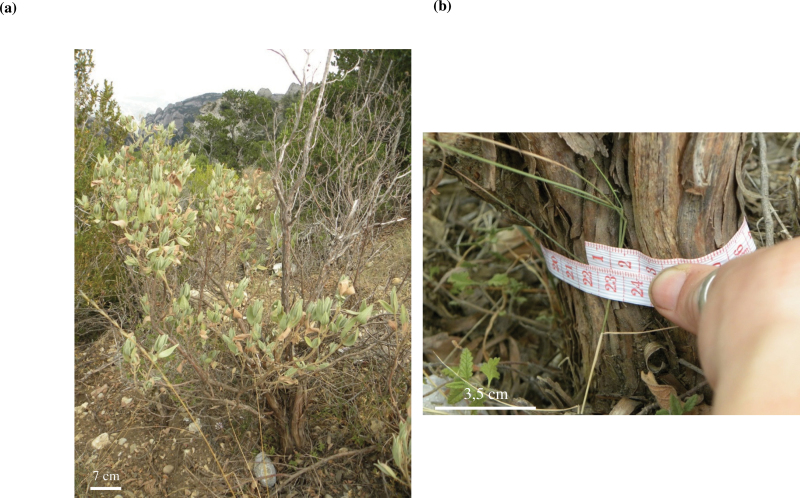
(a) Phenotype of the oldest living individual found in a natural population in the Montserrat Mountains (NE Spain) (perimeter of 22cm). Approximately half of the photosynthetic biomass was lost. (b) Estimation of the perimeter at the base of the trunk. (This figure is available in colour at *JXB* online.)

In the second part of this study, the focus was on confirming these results under natural field conditions. However, plants from a natural population sampled in the Montserrat Mountains (NE Spain) did not show the same symptoms of senescence at the organism level. Seed vigour was not reduced with ageing and, although the number of seeds per fruit decreased with larger perimeters, the seed biomass increased, providing a compensatory mechanism to achieve similar reproductive fitness. Roy and Sonié (1992) found that the annual seed production of 5-year-old *Cistus albidus* and *Cistus monspeliensis* plants was comparable with that of older plants. In addition, the viability and germination capacity were similar in plants of all sizes and the number of aborted seeds was always low and not age dependent. So, what was the difference between the population grown in controlled conditions and one growing naturally? The most likely explanation is the size effect, as can be illustrated using the oldest living individual found in the Montserrat Mountains (Fig. 7), which was 19 years old. This individual comprised a large amount of necromass and a small amount of biomass. Compared with the individuals in the Experimental Fields, which had a large biomass (Fig. 1b), even in the youngest group, the biomass of *C. albidus* growing in the Montserrat Mountains was very low. Note that this pattern was not only found in the oldest individual, but also in other individuals with different ages and trunk perimeters (Supplementary Fig. S6 at *JXB* online). All individuals, including the oldest living *C. albidus* from the Montserrat Mountains, had less biomass than the 3-year-old plants growing in the Experimental Fields. This is probably because plants growing in natural conditions are exposed to more severe environmental conditions, such as extreme droughts and contrasting temperatures (winter/summer). Due to the potential for frost damage, *C. albidus* plants usually have a short stem with three or five branches inserted (Barry, 1960). This pattern was found in some of the sampled plants, especially in older individuals. In natural populations, mortality is greatly influenced by the environment (Picó and Retana, 2008), and *C. albidus* plants usually die due to natural causes (Roy and Sonié, 1992), for example due to competition for water during drought years or biotic causes. Age, size, and growth can also interact with the environment to influence mortality and life span when the environment is stressful (Roach, 2012). Stress also had an effect on the plants in this study as observed with the correlation of the perimeter of the trunk and the age of the plant: individuals growing in natural conditions with similar ages to those in the Experimental Fields had smaller trunks (Supplementary Fig. S5). Furthermore, the oldest living individual found in the Natural Park was 19 years old, while the oldest one found dead was 25 years old. This was the first time that a *C. albidus* older than 15 years old (Roy and Sonié, 1992) has been found. It should be noted that the Montserrat Mountains suffered two important fires, one in 1986 and the other in 1994. The fire of 1986, which occurred 27 years ago, razed much of the forest to the ground. This explains why the oldest individuals are no more than 25 years old.

The *C. albidus* plants growing in the Montserrat Mountains, which are exposed to hard environmental conditions, showed severe biomass loss (including entire branches) and appeared to use their resources to make fewer fruits and seeds but of higher quality. In contrast, in the Experimental Fields, plants produced more fruits and seeds but of lower quality. Although the oldest individuals in the Experimental Fields were 13 years old and were not as old as they could be, the effect of size was apparent. The viability of the seeds was not affected by the perimeter of the trunk in the natural population (Supplementary Table S2 at *JXB* online), but instead was correlated with fruit biomass, seed biomass, and seed biomass per fruit (Fig. 6). Thus the higher the fruit and the seed biomass, the more viable the seed. Furthermore, seed germination capacity was lower than under controlled conditions, suggesting a higher degree of dormancy.

In conclusion, the oldest individuals with a large amount of biomass presented symptoms of senescence at the organism level, as indicated by lower seed production and loss of seed viability, but old individuals that have reduced their size, due to reduced growth and photosynthetic biomass loss, produce seeds of higher quality in natural populations. Such plants are less productive in terms of photosynthetic and seed biomass, but the seeds that they produce are of better quality.

## Supplementary data

Supplementary data are available at *JXB* online.

Figure S1. Germination capacity of seeds of 3-, 8-, and 13-year-old *C. albidus* plants growing in the Experimental Fields.

Figure S2. Gibberellin (GA) content, including that of GA_4_, GA_9_, and GA_24_, in seeds of 3-, 8-, and 13-year-old *C. albidus* plants growing in the Experimental Fields.

Figure S3. Cytokinin content, including that of zeatin (Z), zeatin riboside (ZR), isopentenyladenosine (IPA), dihydrozeatin (DHZ), dihydrozeatin riboside (DHZR), and 2-isopentenyladenine (2-IP), in seeds of 3-, 8-, and 13-year-old *C. albidus* plants growing in the Experimental Fields.

Figure S5. Correlation between plant age and trunk perimeter in *C. albidus* plants growing in the Experimental Fields and Montserrat Mountains.

Figure S6. Comparison of the plant biomass between *C. albidus* from the Experimental Fields and Montserrat Mountains.

Table S1. Fatty acid composition of seeds of 3-, 8-, and 13-year-old *C. albidus* plants growing in the Experimental Fields.

Table S2. Correlation coefficient (*r* ^2^) and *P-*values of Spearman rank correlation analysis between the trunk perimeter and all measured parameters in seeds of *C. albidus* plants growing in a natural population in the Montserrat Mountains (NE Spain).

Supplementary Data

## Abbreviations:

ABA: abscisic acid
GA: gibberellin
IAA: indole-3-acetic acid
JA: jasmonic acid
SA: salicylic acid
Z: zeatin.
