# Supplementary material for: Perennially young: seed production and quality in controlled and natural populations of Cistus albidus reveal compensatory mechanisms that prevent senescence in terms of seed yield and viability
Source: J Exp Bot. 2013 Nov 11;65(1):287–97. doi: 10.1093/jxb/ert372 (PMC3883297; doi:10.1093/jxb/ert372)
Supplement: Supplementary Data [file supp_65_1_287__index.html]

Perennially young: seed production and quality in controlled and natural populations of Cistus albidus reveal compensatory mechanisms that prevent senescence in terms of seed yield and viability — Perennially young: seed production and quality in controlled and natural populations of Cistus albidus reveal compensatory mechanisms that prevent senescence in terms of seed yield and viability — Supplementary Data 

# Perennially young: seed production and quality in controlled and natural populations of *Cistus albidus* reveal compensatory mechanisms that prevent senescence in terms of seed yield and viability

## Supplementary Data

Data files

**Files in this Data Supplement:**

- Supplementary Data - Supplementary Data
