# Supplementary material for: Perennially young: seed production and quality in controlled and natural populations of Cistus albidus reveal compensatory mechanisms that prevent senescence in terms of seed yield and viability
Source: J Exp Bot. 2013 Nov 11;65(1):287–97. doi: 10.1093/jxb/ert372 (PMC3883297; doi:10.1093/jxb/ert372)

Table S1 Fatty acid composition of seeds of 3-, 8- and 13-y-old *C. albidus* plants growing in the Experimental Fields. Data are the mean ± SE of n = 4 individuals with an analysis of 50 mg of seeds per individual. Different letters indicate significant differences between age groups (ANOVA, $P \leq 0.05$).

**Fatty acids**	**Seeds from 3-y-old plants (mg/g DW)**	**Seeds from 8-y-old plants (mg/g DW)**	**Seeds from 13-y-old plants (mg/g DW)**
**Saturated fatty acids**			
**C8:0**	0.015 ± 0.001	0.013 ± 0.002	0.015 ± 0.004
**C10:0**	0.007 ± 0.000	0.006 ± 0.001	0.007 ± 0.000
**C12:0**	0.005 ± 0.000	0.004 ± 0.001	0.005 ± 0.000
**C13:0**	0.008 ± 0.000	0.008 ± 0.001	0.008 ± 0.000
**C14:0**	0.016 ± 0.002	0.016 ± 0.003	0.018 ± 0.001
**C15:0**	0.011 ± 0.001	0.010 ± 0.001	0.012 ± 0.000
**C16:0**	0.617 ± 0.089	0.578 ± 0.149	0.646 ± 0.051
**C16:1**	0.014 ± 0.001	0.013 ± 0.001	0.014 ± 0.001
**C17:0**	0.014 ± 0.001	0.013 ± 0.002	0.015 ± 0.000
**C18:0**	0.125 ± 0.016	0.107 ± 0.030	0.113 ± 0.012
**C20:0**	0.034 ± 0.003	0.032 ± 0.006	0.034 ± 0.002
**C21:0**	0.019 ± 0.001	0.018 ± 0.002	0.020 ± 0.001
**C20:0**	0.029 ± 0.002	0.028 ± 0.005	0.033 ± 0.001
**C23:0**	0.022 ± 0.001	0.020 ± 0.002	0.024 ± 0.001
**C24:0**	0.030 ± 0.001**a**	0.031 ± 0.005**ab**	0.037 ± 0.002**b**
**Total**	**0.969 ± 0.118**	**0.897 ± 0.210**	**1.000 ± 0.078**
**Monounsaturated fatty acids**			
**C18:1n9c**	0.265 ± 0.041	0.234 ± 0.061	0.196 ± 0.024
**C20:1**	0.016 ± 0.001	0.014 ± 0.002	0.015 ± 0.000
**C20:1n9**	0.014 ± 0.001	0.012 ± 0.002	0.014 ± 0.001
**Total**	**0.295 ± 0.042**	**0.260 ± 0.065**	**0.225 ± 0.026**
**Polyunsaturated fatty acids**			
**C18:2n6t**	0.013 ± 0.001	0.012 ± 0.001	0.012 ± 0.001
**C18:2n6c**	1.444 ± 0.249	1.446 ± 0.385	1.539 ± 0.105
**C18:3n3**	0.014 ± 0.000	0.012 ± 0.001	0.013 ± 0.001
**C18:3n6**	0.271 ± 0.043	0.313 ± 0.080	0.306 ± 0.024
**C20:2**	0.021 ± 0.001	0.018 ± 0.002	0.020 ± 0.001
**C20:3n3**	0.011 ± 0.000	0.010 ± 0.001	0.011 ± 0.003
**Total**	**1.775 ± 0.045**	**1.811 ± 0.084**	**1.902 ± 0.028**
**Saturated**	0.969 ± 0.118	0.897 ± 0.210	1.000 ± 0.078
**Monounsaturated**	0.295 ± 0.042	0.260 ± 0.065	0.225 ± 0.026
**Polyunsaturated**	1.775 ± 0.045**a**	1.811 ± 0.084**ab**	1.902 ± 0.028**b**
**Total**	**3.038 ± 0.204**	**2.968 ± 0.358**	**3.127 ± 0.132**

Table S2 Correlation coefficient (r^2) and *P* values (shown in parentheses) of Spearman rank correlation analysis between the trunk perimeter and all measured parameters in seeds of *C. albidus* plants growing in a natural population in the Montserrat Mountains (NE Spain). Numbers in bold indicate significant *P* values (Spearman's rank correlation, $P \leq 0.05$).

Parameter	Trunk perimeter
Fruit biomass (mg FW)	-0.111 (0.169)
Seed biomass (mg FW/fruit)	-0.100 (0.172)
Number of seeds per fruit	-0.201 (**0.028**)
Seed biomass (mg/FW)	0.220 (**0.018**)
% viable	0.068 (0.263)
% dying	-0.014 (0.448)
% aborted	-0.054 (0.305)
α-tocopherol (μg/g seed)	-0.148 (0.097)
β-tocopherol (μg/g seed)	0.011 (0.463)
γ-tocopherol (μg/g seed)	-0.174 (0.063)
α-tocotrienol (μg/g seed)	-0.066 (0.283)
β-tocotrienol (μg/g seed)	-0.102 (0.185)
Total Vitamin E	-0.134 (0.119)
GA_4 (ng/g FW)	0.051 (0.327)
GA_9 (ng/g FW)	-0.071 (0.268)
GA_{24} (ng/g FW)	-0.018 (0.437)
ABA (ng/g FW)	-0.063 (0.290)
IAA (ng/g FW)	0.009 (0.470)
JA (ng/g FW)	-0.090 (0.216)
SA (ng/g FW)	-0.098 (0.196)
2-IP (ng/g FW)	-0.014 (0.452)
DHZ (ng/g FW)	0.043 (0.355)
DHZR (ng/g FW)	-0.145 (0.101)
IPA (ng/g FW)	0.005 (0.482)
Z (ng/g FW)	-0.049 (0.335)
ZR (ng/g FW)	-0.077 (0.249)

Fig. S1 Germination capacity of seeds of 3-, 8- and 13-y-old *C. albidus* plants growing in the Experimental Fields. Data are the mean ± SE of n = 4 individuals with an analysis of 50 seeds per individual. No significant differences were found between any of the three age groups (ANOVA, $P > 0.05$).

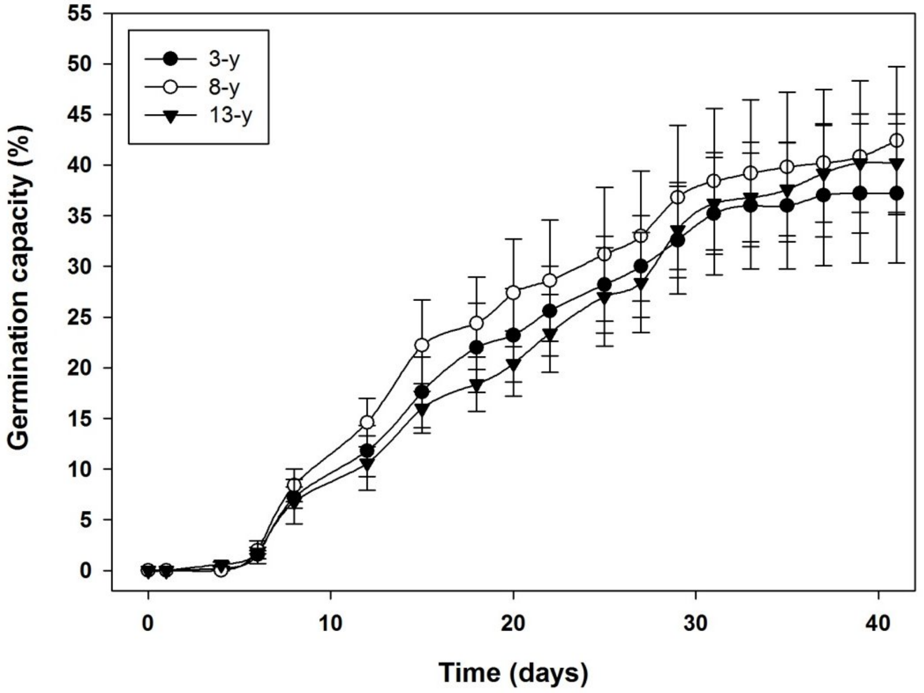

Fig. S2 Gibberellin (GA) content, including that of GA_4, GA_9 and GA_{24}, in seeds of 3-, 8- and 13-y-old *C. albidus* plants growing in the Experimental Fields. Data are the mean ± SE of n = 4 individuals with an analysis of 50 mg of seeds per individual. Different letters indicate significant differences between age groups (ANOVA, $P \leq 0.05$).

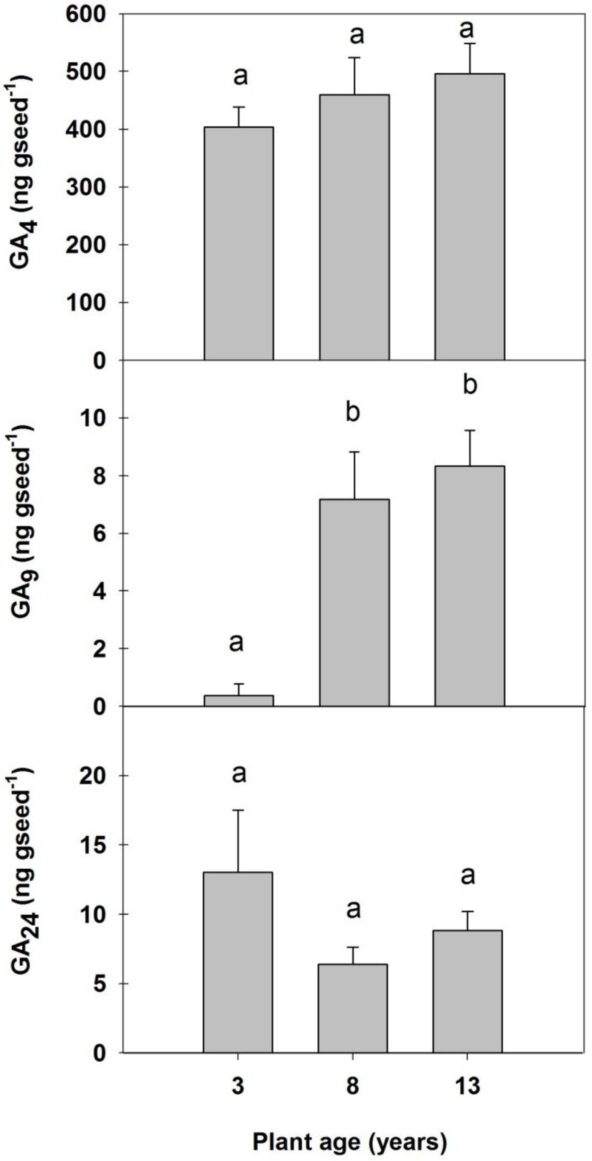

Fig. S3 Cytokinin content, including that of zeatin (Z), zeatin riboside (ZR), isopentenyladenosine (IPA), dihydrozeatin (DHZ), dihydrozeatin riboside (DHZR) and 2-isopentenyladenine (2-IP), in seeds of 3-, 8- and 13-y-old *C. albidus* plants growing in the Experimental Fields. Data are the mean ± SE of n = 4 individuals with an analysis of 50 mg of seeds per individual. Different letters indicate significant differences between age groups (ANOVA, $P \leq 0.05$).

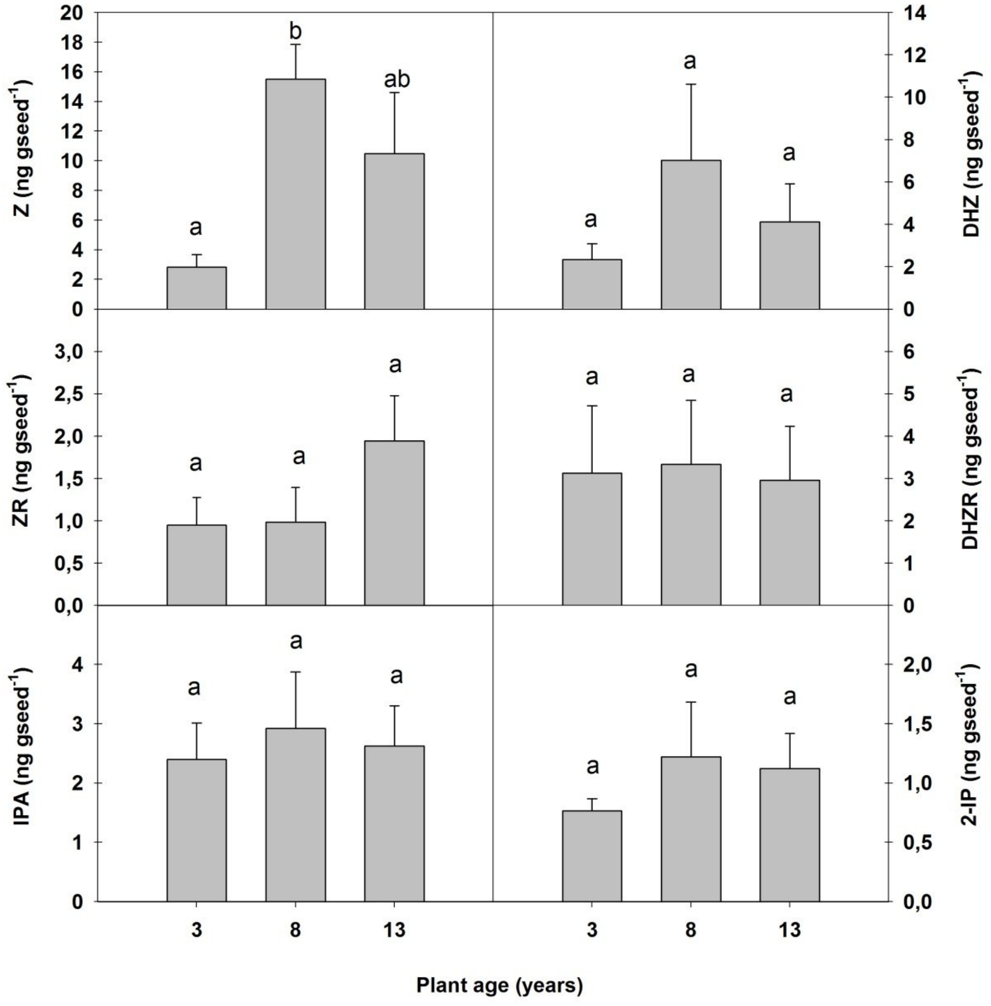

Fig. S4 C and N contents and C/N ratio in seeds of 3-, 8- and 13-y-old *C. albidus* plants growing in the Experimental Fields. Data are the mean ± SE of n = 4 individuals with an analysis of 50 mg of seeds per individual. No significant differences were found between any of the three age groups (ANOVA, $P \leq 0.05$).

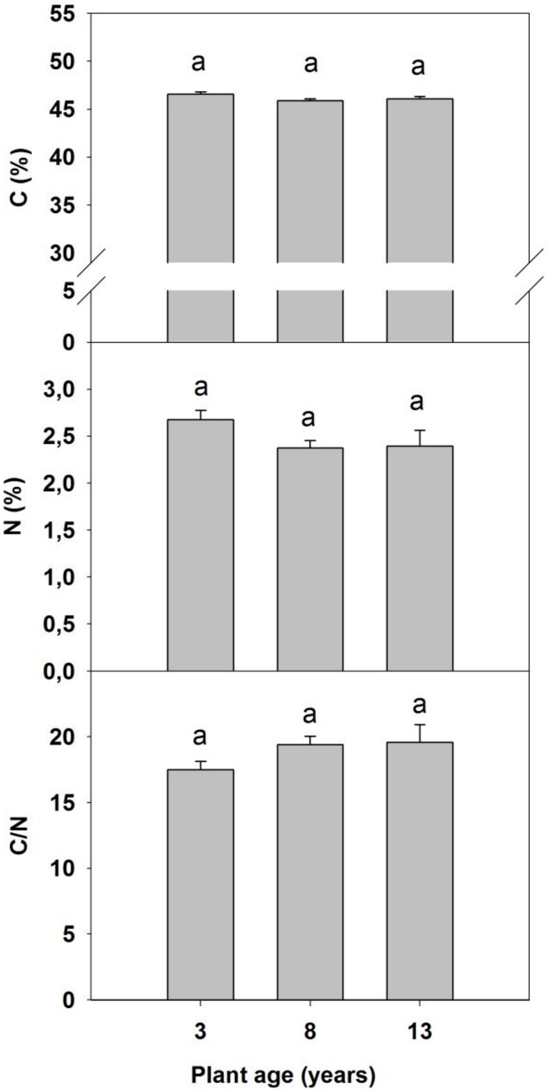

Fig. S5 Correlation between plant age and trunk perimeter in *C. albidus* plants growing in the Experimental Fields (red circles) and Montserrat Mountains (black circles). Spearman's rank analyses showed significant correlations (P<0.001). Note the differences in the slope of the linear regressions.

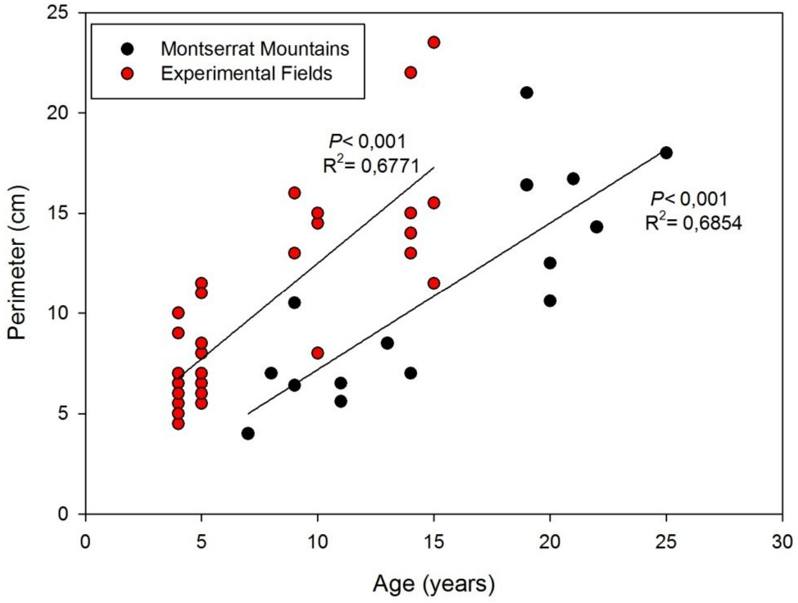

Fig. S6 Comparison of the plant biomass between *C. albidus* from the Experimental Fields and Montserrat Mountains. Examples of trunk rings are also shown. (a–c) Three-, 8 and 13-y-old *C. albidus* plants from the Experimental Fields (from left to right, respectively). (d) Trunk rings from the oldest individual (dead) found in the Montserrat Mountains (25-y-old). (e) Oldest *C. albidus* individual found alive in the Montserrat Mountains (19-y-old). (f–g) Individuals with trunk perimeter of 2.5 and 8 cm in the Montserrat Mountains. (h) Trunk rings from individuals with trunk perimeter of 2.5 and 7 cm, both found in the Montserrat Mountains.

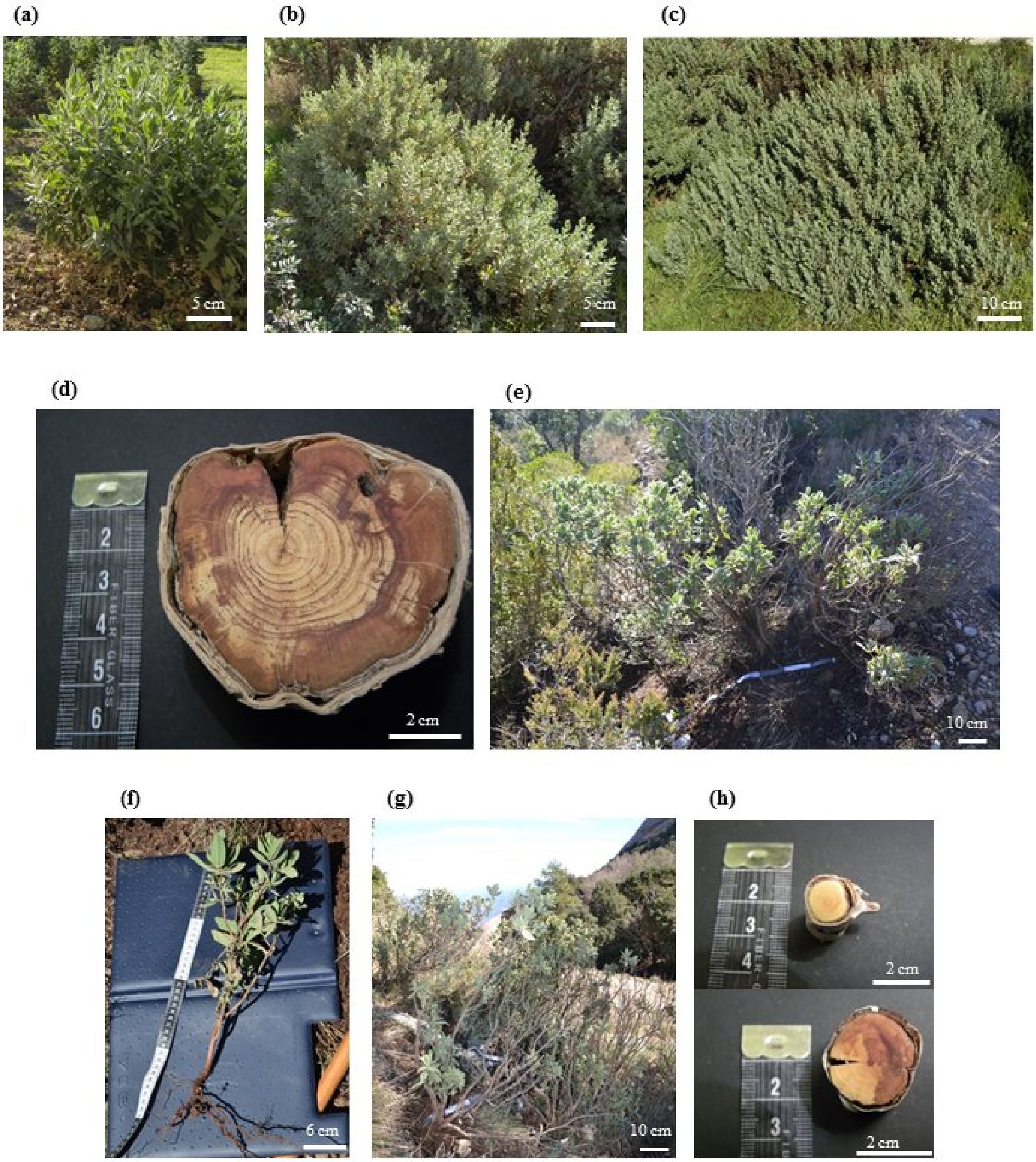

Supplement: Supplementary Data [file supp_ert372_jexbot109587_file001.pdf]
